# Treatment-emergent and trajectory-based peripheral gene expression markers of antidepressant response

**DOI:** 10.1038/s41398-021-01564-8

**Published:** 2021-08-21

**Authors:** Laura M. Fiori, Massimiliano Orri, Zahia Aouabed, Jean François Théroux, Rixing Lin, Corina Nagy, Benicio N. Frey, Raymond W. Lam, Glenda M. MacQueen, Roumen Milev, Daniel J. Müller, Sagar V. Parikh, Susan Rotzinger, Rudolf Uher, Jane A. Foster, Sidney H. Kennedy, Gustavo Turecki

**Affiliations:** 1 McGill Group for Suicide Studies, Douglas Mental Health University Institute, Department of Psychiatry, McGill University, Montreal, Quebec Canada; 2 Department of Psychiatry & Behavioural Neurosciences, McMaster University and St Joseph’s Healthcare Hamilton, Hamilton, Ontario Canada; 3 Department of Psychiatry, University of British Columbia, Vancouver, British Columbia Canada; 4 Hotchkiss Brain Institute, University of Calgary, Calgary, AB Canada; 5 Departments of Psychiatry and Psychology, Queens University, Providence Care Hospital, Kingston, Ontario Canada; 6 Department of Psychiatry, University Health Network, Krembil Research Institute, University of Toronto, Toronto, Ontario Canada; 7 Centre for Addiction and Mental Health, Toronto, Ontario Canada; 8 Department of Psychiatry, University of Michigan, Ann Arbor, Michigan USA; 9 Nova Scotia Health Authority, Halifax, NS Canada; 10 Department of Psychiatry, Dalhousie University, Halifax, Nova Scotia Canada; 11 St Michael’s Hospital, Li Ka Shing Knowledge Institute, Centre for Depression and Suicide Studies, Toronto, Ontario Canada

**Keywords:** Depression, Molecular neuroscience

## Abstract

Identifying biomarkers of antidepressant response may advance personalized treatment of major depressive disorder (MDD). We aimed to identify longitudinal changes in gene expression associated with response to antidepressants in a sample of MDD patients treated with escitalopram. Patients (*N* = 153) from the CAN-BIND-1 cohort were treated for 8 weeks, and depressive symptoms were assessed using the Montgomery-Åsberg Depression Rating Scale at 0, 2, 4, 6, and 8 weeks. We identified three groups of patients according to response status: early responders (22.9%), later responders (32.0%), and nonresponders (45.1%). RNA sequencing was performed in blood obtained at weeks 0, 2, and 8. RNA expression was modeled using growth models, and differences in the longitudinal changes in expression according to response were investigated using multiple regression models. The expression of RNAs related to response was investigated in the brains of depressed individuals, as well as in neuronal cells in vitro. We identified four RNAs (*CERCAM, DARS-AS1, FAM228B, HBEGF*) whose change over time was independently associated with a response status. For all except *HBEGF*, responders showed higher expression over time, compared to nonresponders. While the change in all RNAs differentiated early responders from nonresponders, changes in *DARS-AS1* and *HBEGF* also differentiated later responders from nonresponders. Additionally, *HBEGF* was downregulated in the brains of depressed individuals, and increased in response to escitalopram treatment in vitro. In conclusion, using longitudinal assessments of gene expression, we provide insights into biological processes involved in the intermediate stages of escitalopram response, highlighting several genes with potential utility as biomarkers of antidepressant response.

## Introduction

Major depressive disorder (MDD) is a leading cause of global disease burden. It is estimated to affect more than 300 million people worldwide [1], and to have a lifetime prevalence of 20% [2]. Individuals with MDD are at risk for chronic or recurrent depression, severe functional impairments [3, 4], and suicide [5]. Treating MDD is therefore a public health priority. Antidepressants are widely used to treat MDD [6], yet, response is poor: approximately 60% of patients do not respond to a single trial and 30–40% of patients do not fully respond even after several trials [7]. Additionally, response to antidepressants is highly heterogeneous, and efficacy of the treatment (or lack thereof) may become apparent only after a period of several weeks. This often results in considerable delay in finding the optimal treatment, involving several trials with antidepressants that are ultimately ineffective, and increasing risks and costs.

Significant research efforts are directed toward the identification of biomarkers, measurable before treatment initiation, to help identify individuals who are more likely to respond to antidepressants (i.e., predictor). Evidence to date suggests that several biomarkers reflecting the activity of inflammatory, neurotransmitter, neurotrophic, neuroendocrine, and metabolic systems may be promising predictors of antidepressant response [8]. However, translation of these findings into clinical guidelines to select the appropriate antidepressant has proven to be difficult [9]. Treatment-emergent (i.e., mediator) biomarkers provide alternative information that is equally valuable as they are helpful to better understand mechanisms involved in antidepressant treatment response. While predictor biomarkers may be useful in the initial treatment selection, treatment-emergent biomarkers can be used as indices of antidepressant efficacy during the course of treatment [10]. In clinical practice, such biomarkers can inform clinicians to change an ineffective treatment to another that is more likely to be effective within a few weeks of treatment initiation, thus reducing the number of ineffective antidepressant treatment trials, and shorten the time to discover the best treatment.

Gene expression measured in the blood is easily accessible in patients undergoing antidepressant treatment, and might potentially be used to inform clinical decisions. Gene expression measured peripherally may not necessarily reflect gene expression in brain cells. However, MDD is a systemic illness, and treatment-induced molecular changes are likely to result in changes in gene expression observable in peripheral tissues [11]. To date, the majority of studies investigating the relationship between the gene expression and antidepressant response have focused on either baseline gene expression levels, or have assessed changes in expression between the baseline and the completion point of a trial (typically 8 weeks). By not investigating intermediate stages in response, these studies are not able to identify gene expression biomarkers, which could be used to inform early treatment decisions nor are they able to detect gene expression trajectories associated with antidepressant response or lack thereof.

The objective of this study was to identify longitudinal changes in peripheral gene expression associated with response to antidepressant pharmacotherapy in a sample of patients treated with escitalopram. We relied on repeated measures of clinical and molecular data collected at baseline and after 2 and 8 weeks of antidepressant treatment in a sample of patients enrolled in a multicenter Canadian study. Genes related to antidepressant response were further characterized in postmortem human brain and in neuronal cell cultures.

## Materials and methods

### Sample and clinical assessment

We used data from the CAN-BIND-1 study, described in detail [12, 13]. Participants between 18 and 61 years of age suffering from MDD, who scored 21 or more on the Montgomery-Åsberg Depression Rating Scale (MADRS) [14] were recruited from physician referrals or advertisements. Recruitment took place between August 2013 and December 2016 at six academic centers in Canada. Exclusion criteria included bipolar disorder, high suicidal risk, psychosis, drug dependency, pregnancy or breastfeeding, and failure to respond after four or more adequate pharmacologic interventions in the current episode or to a previous trial of escitalopram. At baseline, the Mini-International Neuropsychiatric Interview [15] Version 6.1 was administered to confirm or rule out MDD status and to assess the presence of other psychiatric comorbidities. A total of 211 eligible participants were treated with escitalopram (10–20 mg) for 8 weeks. After baseline assessment (T0), recruited patients were reassessed every 2 weeks (T2–T8) with the MADRS. Additionally, blood samples were collected at T0, T2, and T8 for molecular analyses. We also included sex- and age-matched healthy controls (HC, N = 104) [12, 13]. At T0, RNA sequencing data were available for 104 HC and 201 patients. The trajectory analysis is based on a sample of 153 patients with available data on both the MADRS and RNA sequencing data at all three timepoints. All participants provided written informed consent, and ethics approval was obtained at each center. The trial was registered at ClinicalTrials.gov (identifier: NCT01655706).

### Response to antidepressant treatment

Response status at T8 was determined by calculating the ratio of MADRS at T8 relative to T0 (∆MADRS). Participants showing a 50% reduction of their MADRS scores were considered responders to escitalopram treatment (*N* = 84, 54.9%), those who did not were considered nonresponders (*N* = 69, 45.1%).

### RNA sequencing

Whole blood for RNA was collected in EDTA tubes and filtered using LeukoLOCK filters (Life Technologies). Total RNA was extracted from leukocytes using a modified version of the LeukoLOCK Total RNA Isolation System protocol, and included DNase treatment to remove genomic DNA. RNA quality was assessed using the Agilent 2200 Tapestation, and only samples with RNA Integrity Number (RIN) ≥ 6.0 were used. All libraries were prepared using the Illumina TruSeq mRNA stranded protocol following the manufacturer’s instructions. Samples were sequenced at the McGill University and Genome Quebec Innovation Centre (Montreal, Canada) using the Illumina HiSeq4000 with 100nt paired-end reads. FASTXToolkit and Trimmomatic were respectively used for quality and adapter trimming. Tophat2, using bowtie2 was used to align the cleaned reads to the reference genome. Reads that lost their mates through the cleaning process were aligned independently from the reads that still had pairs. Quantification on each gene’s expression was estimated using HTSeq-count and a reference transcript annotation from ENSEMBL. Counts for the paired and orphaned reads for each sample were added to each other. Normalization was conducted on the resulting gene matrix using DESeq2. All RNA expression values were log2 transformed for data analysis and adjusted for age, gender, and RIN.

### Longitudinal analysis

#### Identifying trajectories of antidepressant response

Data on MADRS scores between T0 and T8 were modeled using a longitudinal *k*-mean algorithm to identify clusters of individuals based on the evolution over time of their scores [16]. This procedure assigns participants who are homogeneous in their MADRS score evolution to the same trajectory. The best model is selected based on both the statistical criteria (e.g., Calinski & Harabasz criterion) [16] and interpretability of the clustering solution.

#### Selecting target RNAs for longitudinal analyses

From the initial pool of sequenced RNAs, we identified RNAs for subsequent longitudinal analyses based on the following stepwise criteria: 1) RNA must show differential expression between the MDD patients and HC at T0. This was assessed using the general linear model (GLM) implementation of DESeq2, including sex, age, and RIN as covariates and selecting the RNAs that were differentially expressed between the groups after applying a False Discovery Rate (FDR) correction of 20%; 2) Remaining RNAs must show a correlation with the change in MADRS at T8. The change in MADRS between the T0 and T8 (∆MADRS) was calculated as a ratio, and a Pearson correlation (*P* < 0.05) was used to relate RNA to ∆MADRS (*P* < 0.05). 3) Remaining RNAs cannot correlate with ∆MADRS at T0 (*P* < 0.2). This strict cut-off was chosen because our aim was to investigate differences in RNA expression over time that were not identifiable at baseline.

#### Modeling longitudinal changes in RNA expression

We modeled the evolution over time (i.e., trajectory) of each selected RNA by fitting growth models using the R package *lavaan*. This procedure, based on structural equations modeling (SEM), allowed us to use the repeated measure of each RNA to describe their linear change over time using two random-effect parameters: intercept, describing the level of an RNA at baseline (T0), and slope, describing the rate of change between T0 and T8. The fit of each growth model was assessed using the chi-square statistics (a nonsignificant chi-square suggesting good fit), the Comparative Fit Index (CFI), the Tucker Lewis index (TLI), the Goodness of Fit Index (GFI; all indices indicated good fit if >0.90), the Root Mean Square Error of Approximation (RMSEA), and the Standardized Root Mean Square Residual (SRMR, both good fit if <0.8).

#### Identifying differences in longitudinal changes in RNA expression according to response status

We performed this analysis in three steps. First, for each RNA, we tested the association between the change in RNA over time and response to escitalopram at T8 (yes/no) using a binary logistic regression model with intercept and slope parameters as predictors of response status. Second, the RNAs associated with response to escitalopram (*P* < 0.05) were jointly modeled using a multivariate logistic regression to estimate their independent association with response to escitalopram. Third, the same RNAs were jointly modeled using a multivariable multinomial regression to estimate their independent association with early or later response. Multivariable regressions were adjusted for self-reported gender (male/female), age (continuous variable), history of any anxiety disorders (i.e., agoraphobia, generalized anxiety disorder, post-traumatic stress disorder, social phobia, panic disorder as assessed with the MINI), history of suicidality (assessed with the MINI), and a FDR of 5% was used to take into account multiple comparisons. For all analyses, a log2 transformation was used to account for the non-normal distribution of RNA expression variables.

### Anterior cingulate cortex (ACC) gene expression

#### Brain samples

Gene expression in the ACC was previously described in [17]. Briefly, ACC tissue was obtained from the Douglas-Bell Canada Brain Bank from subjects who died suddenly without prolonged agonal state or medical illness, and with no psychiatric history (controls, *N* = 24); and subjects who died by suicide in the context of a major depressive episode and had a history of severe child abuse (MDD, *N* = 26). Psychological autopsies were performed as described previously [18], based on DSM-IV criteria. Antidepressant use was determined using 3-month history of antidepressant prescriptions, as well as toxicology reports at the time of death. Written informed consent was obtained from next-of-kin. This study was approved by the institutional review board of the Douglas Mental Health University Institute.

#### RNA sequencing

All RNA sequencing and data processing steps were performed as above, with the exception of the use of an Illumina HiSeq2000 for library sequencing.

#### Statistical analysis

Sequencing data were log2 transformed for analyses. Two-tailed *t* tests were performed to assess differences between cases and controls. One-way ANOVAs, with post hoc Tukey’s test for multiple comparisons, were used to assess the effects of antidepressant history.

### In vitro gene expression

#### Cell culture

Hindbrain neuronal progenitor cells (NPCs) were generated from human induced pluripotent stem cells (iPSCs), using a protocol adapted from [19]. Human iPSCs were first cultured in DMEM/F12 (Gibco) supplemented with N2 (Gibco), B27 (Gibco), nonessential amino acids (Gibco), 1% GlutaMAX (Gibco), 2 µM SB431542 (STEMCELL Tech.), 2 µM DMH1 (Tocris), and 3 µM CHIR99021 (Tocris); collectively referred to as SDC media. Culturing in SDC media for 1 week induced human iPSC differentiation into rostral hindbrain neural stem cells (NSCs). Rostral hindbrain NSCs colonies were selected and re-plated in SDC media supplemented with 1000 ng/ml of SHH C25II (GenScript). Ventral rostral hindbrain NSC colonies were collected and re-plated in SDC + SHH media along with 10 ng/ml of FGF4 (Pepro Tech). Growth in SDC + SHH + FGF4 media-induced ventral rostral hindbrain NSC differentiation into hindbrain NPCs after 1 week. Hindbrain NPCs were GBX2, HOXA2, and HOXA4 positive as assessed via PCR to confirm hindbrain specificity at this developmental stage. All cells were grown in a 5% CO_2_ humidified incubator at 37 °C.

#### Drug Treatments

NPCs were cultured in 24 well plate and differentiated, for 2 weeks, into neuron-like cells in neurobasal media (Gibco) supplemented with N2, B27, NEAA, 1 µg/ml laminin (Sigma), 0.2 mM vitamin C (Sigma), 2.5 µM DAPT (Sigma), 10 ng/ml GDNF (GenScript), 10 ng/ml BDNF (GenScript), 10 ng/ml insulin-like growth factor-I (Pepro Tech), and 1 ng/ml transforming growth factor β3 (Pepro Tech). Following 2 weeks of differentiation, culture media was supplemented with escitalopram (Sigma-Aldrich, E4786; 100 µM), duloxetine (Sigma-Aldrich, Y0001453; 10 µM), imipramine (Sigma-Aldrich, I7379; 10 µM), haloperidol (Sigma-Aldrich, H1512; 10 µM), lithium (Sigma-Aldrich, L4408; 1 mM), or left untreated (controls). Cells for each drug treatment were incubated for 48 h before harvest and RNA extractions. Each drug treatment was performed in triplicate. RNA was extracted using the Zymo DirectZol RNA Extraction kit. RNA was reverse-transcribed using M-MLV Reverse Transcriptase (200 U/µL) (ThermoFisher) with random hexamers. RT-PCR was performed using SYBR green (Applied Biosystems). Reactions were run in triplicate using the QuantStudio 6 Flex System and data collected using QuantStudio Real-Time PCR Software v1.3. Expression levels were calculated using the relative (2^-ΔΔCt^) quantification method, with B-actin and GAPDH as endogenous controls. Results are presented using B-actin as the endogenous control; however, values were highly correlated between B-actin and GAPDH (not shown). Primer sequences are shown in Supplemental Table S1.

#### Statistical Analysis

One-way ANOVAs, with post hoc Dunnett’s test for multiple comparisons, were used to assess the effects of drug treatments relative to untreated cells.

## Results

The demographic characteristics and response status of the 153 participants in the study are presented in Table 1. Participants were on average 36-year-old, 62.7% were female, and 79% were Caucasian. Half of the participants reported a history of comorbid anxiety disorders (49.7%). Differences in demographic and clinical characteristics across response status were not statistically significant.

**Table 1:** Demographic and clinical characteristics of the sample.

	Whole sample (*N* = 153)		By response status			
			NR (*N* = 69, 45.1%)	LR (*N* = 49, 32.0%)	ER (*N* = 35, 22.9%)	*P*
Demographic characteristics						
Age, years	35.90 (12.48)		36.12 (13.05)	36.55 (11.90)	34.57 (12.39)	0.762
Female sex	96 (62.7)		41 (59.4)	29 (59.2)	26 (74.3)	0.274
Years of education	16.92 (2.17)		16.86 (2.12)	16.96 (2.43)	17.00 (1.94)	0.941
Married/cohabitating	86 (56.2)		35 (50.7)	30 (61.2)	21 (60.0)	0.461
Caucasian ethnicity	108 (79.4)		49 (79.0)	35 (77.8)	24 (82.8)	0.870
Clinical characteristics						
History of anxiety disorders	76 (49.7)		30 (43.5)	28 (57.1)	18 (51.4)	0.334
Agoraphobia	17 (11.2)		8 (11.6)	4 (8.2)	5 (14.7)	0.642
GAD	35 (22.9)		19 (27.5)	8 (16.3)	8 (22.9)	0.360
OCD	7 (4.6)		4 (5.8)	1 (2.0)	2 (5.7)	0.588
PD	23 (15.0)		9 (13.0)	8 (16.3)	6 (17.1)	0.819
PTSD	11 (7.2)		5 (7.2)	3 (6.1)	3 (8.6)	0.912
SP	34 (22.2)		10 (14.5)	19 (38.8)	5 (14.3)	0.003
History of suicidality	115 (75.2)		47 (68.1)	41 (83.7)	27 (77.1)	0.149

Characteristics are described as N and %, except for age and years of education (described using mean and standard deviation). Comparison by the group is based on Chi-square or Fisher exact test for categorical variables, and on ANOVA for continuous variables.

*ER* early responders, *GAD* generalized anxiety disorder, *LR* later responders, *NR* nonresponders, *OCD* obsessive-compulsive disorder, *PD* panic disorder, *PTSD* post-traumatic stress disorder, *SP* social phobia

**Fig. 1:**
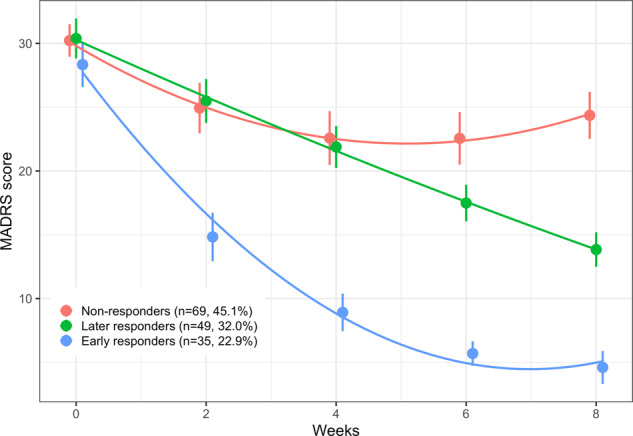
Trajectories of changes over time in MADRS score. Observed mean of the Montgomery-Åsberg Depression Rating Scale (MADRS) score according to treatment status determined at T8. Responders are those showing a 50% reduction in MADRS score at T8, of which we distinguished early responders (50% reduction in MADRS score criterion already met at T2), and later responders (50% reduction in MADRS score criterion met after T2).

Based on biweekly MADRS scores reported from T0 to T8, two groups of responders and two groups of nonresponders were identified: (1) early responders, already reaching the 50% reduction of MADRS score at T2 (*N* = 35, 22.9%), (2) later responders, reaching the 50% reduction of MADRS after T2 (*N* = 49, 32.0%); (3) nonresponders1, showing little/no reduction of MADRS score at any time point (*N* = 46, 30.1%); (4) nonresponders2, initially showing a decrease in MADRS score similar to that of early responders but subsequently showing an increase in MADRS score (*N* = 23, 15.0%). For the purposes of this analysis, we merged these two groups of nonresponders (Fig. 1).

We started with an initial pool of 24, 428 RNA transcripts from leukocyte samples and ended up selecting 32 transcripts for further analysis. First, we retained the 2502 transcripts showing differential expression at baseline between the depressed patients and controls (Supplemental Table S2). Of those, 90 were excluded due to missing data at either T0 or T8. Of the remaining 2412 transcripts, we identified 62 associated with ∆MADRS score at T8. Finally, we excluded 30 RNAs that correlated with ∆MADRS at T0 resulting in 32 RNAs for the longitudinal analysis (Supplemental Figure S1).

**Fig. 2:**
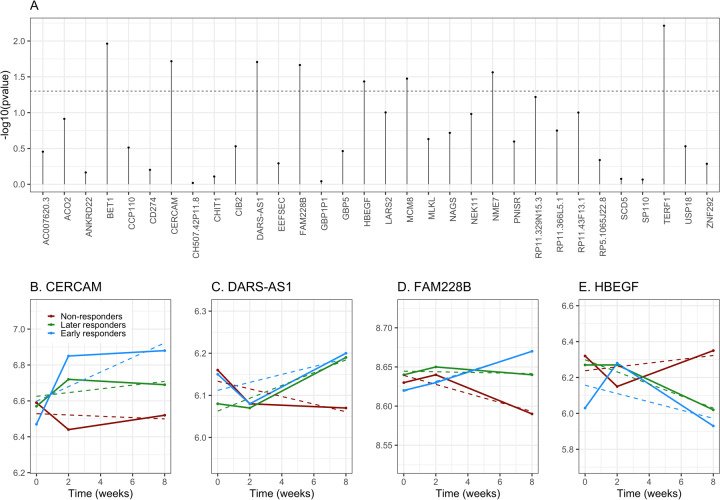
Relationship between change over time and response to escitalopram treatment. **A** Association between change over time (slope) of the 32 selected RNAs and response to escitalopram treatment at T8. The *p* values (log10 transformed, *y*-axis) for the association between response status and RNA slope are shown for each of the 32 selected RNAs (*x*-axis). The dotted line represents the threshold of *P* < 0.05 used to select RNAs for the next analysis steps. **B–E** Change over time of the 4 significant RNAs by response to escitalopram treatment. Expression trajectories over time are shown for CERCAM **B**, DARS-AS1 **C**, FAM228B **D**, and HBEGF **E** among individuals classified as early responders, later responders, and nonresponders. Solid lines and points represent observed means, dashed lines represent linear slopes.

Using growth models, we estimated the trajectory for all 32 RNAs (i.e., their evolution over time); all models showed a good fit to the data (Supplemental Table S3). Of these 32 RNAs, we identified 8 whose change in expression over time (slope) was different across responders and nonresponders (*P* < 0.05; Fig. 2A): Bet1 golgi vesicular membrane trafficking protein (*BET1*), cerebral endothelial cell adhesion molecule (*CERCAM*), DARS1 antisense RNA 1 (*DARS-AS1*), family with sequence similarity 228 member B (*FAM228B*), heparin-binding EGF-like growth factor (*HBEGF*), minichromosome maintenance 8 homologous recombination repair factor (*MCM8*), NME/NM23 family member 7 (*NME7*), and telomeric repeat binding factor 1 (*TERF1*).

Then, we jointly assessed the association between changes in expression of these eight RNAs and response status to investigate their independent association with treatment response using a multinomial logistic regression. After controlling for multiple testing, we found four RNAs independently predicting response status: *CERCAM*, *DARS-AS1*, *FAM228B*, *and HBEGF* (Table 2). For all except *HBEGF*, responders showed higher expression over time, compared to nonresponders (Fig. 2, B–E).

**Table 2:** Association between changes in RNA expression and risk response status.

RNA	log(RR)	SE	*P*	FDR	RR
Responders vs. nonresponders					
BET1	0.776	0.471	0.100	0.159	2.17 (0.86-5.47)
CERCAM	1.064	0.323	0.001	0.023*	2.90 (1.54-5.46)
DARS-AS1	−0.817	0.387	0.035	0.076	0.44 (0.21-0.94)
FAM228B	1.079	0.430	0.012	0.041*	2.94 (1.27-6.83)
HBEGF	0.748	0.340	0.028	0.066	2.11 (1.09-4.11)
MCM8	0.563	0.303	0.063	0.116	1.76 (0.97-3.18)
NME7	0.259	0.302	0.392	0.450	1.30 (0.72-2.34)
TERF1	0.095	0.382	0.803	0.803	1.10 (0.52-2.32)
Early vs. nonresponders					
BET1	0.659	0.368	0.073	0.125	1.93 (0.94-3.97)
CERCAM	0.794	0.260	0.002	0.023*	2.21 (1.33-3.68)
DARS-AS1	−0.879	0.309	0.004	0.023*	0.42 (0.23-0.76)
FAM228B	0.814	0.340	0.017	0.050*	2.26 (1.16-4.40)
HBEGF	0.799	0.282	0.005	0.023*	2.22 (1.28-3.87)
MCM8	0.333	0.244	0.173	0.244	1.40 (0.86-2.25)
NME7	0.297	0.240	0.217	0.289	1.35 (0.84-2.15)
TERF1	0.211	0.296	0.476	0.497	1.24 (0.69-2.21)
Later vs. nonresponders					
BET1	0.632	0.396	0.110	0.165	1.88 (0.87-4.09)
CERCAM	0.647	0.287	0.024	0.065	1.91 (1.09-3.35)
DARS-AS1	−0.920	0.342	0.007	0.028*	0.40 (0.20-0.78)
FAM228B	0.695	0.371	0.061	0.116	2.00 (0.97-4.15)
HBEGF	0.888	0.315	0.005	0.023*	2.43 (1.31-4.51)
MCM8	0.228	0.267	0.394	0.450	1.26 (0.74-2.12)
NME7	0.296	0.266	0.265	0.335	1.34 (0.80-2.26)
TERF1	0.244	0.328	0.456	0.497	1.28 (0.67-2.43)

The table reports the estimates from a multivariable multinomial regression model predicting response status (i.e., dependent categorical variable: early, later, and nonresponders; nonresponders used as base category) from longitudinal changes in RNAs, independently from one another. Risk ratios (RR) increase in the likelihood of being an early responder (or later responder) vs. nonresponder for 1 standard deviation increase in RNA slope. The model is adjusted for age, sex, anxiety disorders, and suicidality.

*SE* standard error, *RR* risk ratio, *FDR* false discovery rate.

*RNA with FDR < 0.05.

**Fig. 3:**
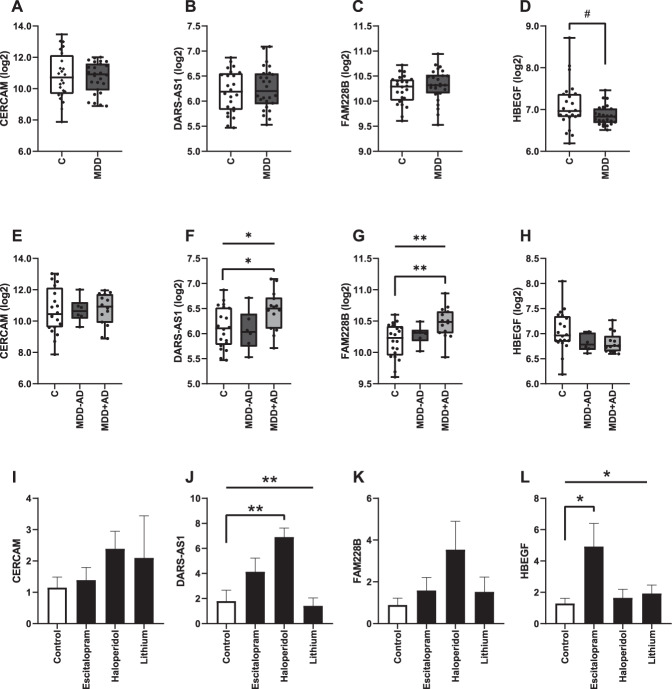
Expression in the brain and in vitro. Gene expression in the anterior cingulate cortex of controls **C**, individuals with major depressive disorder (MDD), depressed individuals not taking antidepressants (MDD − AD), and depressed individuals taking antidepressants (MDD + AD) was quantified by RNA sequencing, for CERCAM **A**, **E**, DARS-AS1 **B**, **F**, FAM228B **C**, **G**, and HBEGF **D**, **H**. The effects of drug treatment on gene expression in neural progenitor cells was assessed after 48 h of treatment, for CERCAM **I**, DARS-AS1 **J**, FAM228B **K**, and HBEGF **L**. For cell experiments, expression values were normalized to B-actin, and error bars represent SEM.

We next assessed the expression of these four genes in the brains of subjects who met the criteria for MDD, as previously published [17] (Fig. 3, A–D), and found a trend for decreased expression of *HBEGF* in depressed individuals (*P* = 0.065). Furthermore, we examined the effects of antidepressant treatment on expression, by dividing the MDD group into those who were taking antidepressants and those with no history or positive toxicology for antidepressants (Fig. 3, E-H). This analysis identified elevated expression in individuals taking antidepressants relative to controls for both the *DARS-AS1* (ANOVA *P* *=* 0.04, Tukey’s *P* = 0.05) and *FAM228B* (ANOVA *P* *=* 0.009, Tukey’s *P* = 0.007).

Finally, we examined the effects of drug treatment on the expression of these four genes in vitro, using a hindbrain neuronal cell culture (Fig. 3, I–L). Cells were treated with three different classes of psychotherapeutic agents in order to assess the specificity of gene expression changes. We found increased expression of *HBEGF* in cells, which were treated with escitalopram (ANOVA *P* *=* 0.05, Dunnett’s *P* = 0.04), with no significant differences when treated with haloperidol or lithium. In complementary analyses, we found that this effect was specific to escitalopram and not to other antidepressants such as duloxetine or imipramine (Supplemental Figure S2). No other genes were significantly altered by escitalopram treatment. Interestingly, *DARS-AS1* displayed significantly elevated expression in cells treated with haloperidol and imipramine (ANOVA *P* *=* 0.002, Dunnett’s *P* = 0.004 and 0.005, respectively).

## Discussion

In this study, we used longitudinal gene expression data to identify RNAs displaying differential patterns of expression, which were related to trajectories of clinical response to escitalopram treatment. To this end, we identified three patterns of clinical response, based on the MADRS score, which differed in both the timing of changes in depressive symptoms, as well as response status at the end of the trial. These trajectories of response were associated with changes of expression of four genes across time and build upon previous reports on clinical trajectories of antidepressant response [20, 21].

The best-characterized gene among these four is HBEGF. This gene belongs to the epidermal growth factor family, and preferentially binds both the epidermal growth factor receptor (EGFR) and erb-b2 receptor tyrosine kinase 4 (ERBB4) [22]. Both of these receptors have previously been implicated in psychiatric disorders, including schizophrenia, mood disorders, and antidepressant response [23–29]. Furthermore, dysregulation of HBEGF has been implicated in depression [30], and mice in which HBEGF have been knocked out display behavioral phenotypes, which are responsive to antipsychotics as well as altered dopamine and serotonin levels in the brain [31]. In addition to its relationship with escitalopram response, we also found it to be differentially expressed in the brain in individuals with depression who died by suicide, suggesting that our findings in the blood may also indicate central effects. Moreover, the expression of this gene was altered by escitalopram treatment in vitro, further supporting a direct role for HBEGF in antidepressant response.

Unlike HBEGF, the function of CERCAM has not been well-studied. CERCAM was previously known as glycosyltransferase 25 family member 3 but does not display beta-galactosyltransferase activity in vitro [32]. Instead, one study identified it to be a cell adhesion protein involved in the migration of leukocytes across the blood-brain barrier [33]. This is particularly interesting given the relationship between blood-brain barrier integrity and depressive phenotypes [34], as well as consistent findings of increased inflammation in depressive disorders [35–37]. Furthermore, CERCAM displays enriched expression in the brain and pituitary gland (gtexportal.org), suggesting this protein has important neurological functions.

To date, no function of FAM228B has been identified. However, this gene is ubiquitously expressed across most tissues, including the brain, likely indicating functional importance. Additionally, it demonstrated altered expression in the brains of depressed individuals who were being treated with antidepressants, suggesting a potential relationship between our peripheral and central findings. Future studies will be needed in order to identify the function of this gene.

Unlike the protein-coding genes above, DARS-AS1 is a long noncoding RNA (lncRNA). The majority of studies investigating this lncRNA have been related to its role in cancer, where it acts to regulate the expression of several microRNAs, including miR-129, miR-194-5p, miR-532-3p, and miR-628-5p [38–41], as well as the protein RNA-binding motif protein 39 (RBM39) [42]. As a number of studies have highlighted the importance of microRNAs in antidepressant response [43, 44], it seems plausible that the role of DARS-AS1 in escitalopram response involves modulation of microRNA expression. Similar to FAM228B, this gene was altered in the brains of antidepressant-treated individuals, supporting a role for its involvement in treatment response.

Comparable to depression, the antidepressant response is highly heterogeneous and involves complex interplays among numerous signaling pathways. Similarly, the neurobiological changes that occur during antidepressant treatment are unique to each individual, based on their underlying biology and environmental factors. As such, both the biological effects of different pathways and timing of these effects during the course of treatment, can vary greatly between individuals. At the clinical level, these differences are apparent at both the level of depressive symptoms, as well as the trajectory of symptoms over time. Reflecting this, we identified several groups of response trajectories among participants in this study. By incorporating response trajectories into our analyses, we were able to identify genes whose longitudinal patterns of expression were related to response. Differences in longitudinal gene expression between responders and nonresponders were similar regardless of the early vs. later response pattern to antidepressant treatment. This suggests that these genes are robust biomarkers of antidepressant response independently from the rate of improvement of the depressive symptoms. For example, changes over time in these genes’ expression may suggest future adequate response to antidepressant even if drastic clinical improvements are not yet observed. Conversely, lack of changes over time of these genes’ expression may suggest the absence of future antidepressant response, if clinical symptoms are also not improving. Importantly, in the present study, we chose to focus on genes whose expression was not different between the groups at baseline, as our objective was to identify genes that mediate, rather than predict, antidepressant response. By doing so, we were able to identify genes that may be used to more precisely monitor antidepressant response, or may represent potential new antidepressant treatment targets. Most large-scale gene expression studies investigating antidepressant response over time focus on expression differences between two time points, typically prior to treatment initiation, as well as after a standard course of treatment (generally eight weeks). Although valuable, these studies lack precision, as they fail to include intermediate time points, which are often clinically relevant [45, 46]. Indeed, as the molecular changes underlying depressive symptoms are also likely to display temporal differences similar to clinical changes, vital information can be gained by analysing gene expression at additional time points during treatment.

Our study has numerous strengths, including (1) the use of a large sample of patients treated with escitalopram in a well-characterized multicenter cohort, (2) high-throughput RNA sequencing data for all patients at three clinically relevant time points during treatment, and (3) the use of longitudinal models which allowed us to identify patterns of antidepressant response, as well as to study the longitudinal course of gene expression during antidepressant treatment. However, the study has some limitations. First, although we analyzed a moderately large sample, statistical power may have been limited for some comparisons, especially taking into account corrections for multiple testing. This may have led to conservative results (i.e., failure to identify additional genes associated with response). Second, although we used longitudinal data on RNA expression, reliance on three time points only allowed us to model linear changes over time. Studies with larger samples and additional data points are needed to replicate our results and to enable a fine-grained examination of longitudinal patterns of gene expression (e.g., quadratic trajectories). Finally, it must be noted that while the genes we identified were associated with response, our analyses cannot infer causality, such that it is not clear if these genes are involved in clinical response, or if they are downstream to pathways, which are affected by the response. Additionally, the present study was performed using peripheral blood samples, and the relationship between the gene expression in the blood and brain is unclear. However, we identified differential expression of HBEGF, DARS-AS1, and FAM228B in the brains of depressed individuals who died by suicide, suggesting that these gene expression patterns in the blood reflect biological processes occurring in the brain. More detailed analyses, including antidepressant trials with additional time points, as well as animal studies in which gene expression can be experimentally manipulated, will be necessary to better elucidate the role of these genes in antidepressant response.

In conclusion, in a large cohort of escitalopram-treated patients with MDD, we identified patterns of treatment response, which were individually associated with changes in the expression patterns across time of four genes. Our study provides greater insight into biological processes, which are involved in the intermediate stages of escitalopram the response, and highlights several genes whose roles in antidepressant response had not been previously identified. Future work will be needed to more thoroughly characterize the role of these genes and their related pathways.

## Supplementary information

Supplemental Material

Supplemental Table 2
