# Supplementary material for: Treatment-emergent and trajectory-based peripheral gene expression markers of antidepressant response
Source: Transl Psychiatry. 2021 Aug 21;11:439. doi: 10.1038/s41398-021-01564-8 (PMC8380246; doi:10.1038/s41398-021-01564-8)
Supplement: Supplementary file 1 — Supplemental Material [file 41398_2021_1564_MOESM1_ESM.docx]

**Supplementary material**

**Table S1.** Primers used for quantitative RT-PCR.

| **Primer** | **Sequence** |
| --- | --- |
| CERCAM-F1 | TCTTCGCCTATGCCTGCCAG |
| CERCAM-R1 | GACCCTCTCGTCTTCCAGCC |
| DARS-AS1-F2 | GTCCCTAACAGAGTGGTGAGG |
| DARS-AS1-R2 | CAGCTTGGGCTTCATACAGGA |
| FAM228B-F2 | GCTAAAGAAGATACTGAGGCAGC |
| FAM228B-R2 | GCCACACAGTCAACCCATCT |
| HBEGF-F1 | CATCTGTCTGTCTGCTGGTCATC |
| HBEGF-R1 | GGGAATTAGTCATGCCCAACTTCAC |
| Bactin-F | AAGACCTGTACGCCAACACA |
| Bactin-R | GCAGTGATCTCCTTCTGCATC |
| GAPDH-F | TTGTCAAGCTCATTTCCTGG |
| GAPDH-R | TGTGAGGAGGGGAGATTCAG |

**Table S3.** Fit indices of the growth models

| RNA | Chi-square | CFI | TLI | GFI | RMSEA | SRMR |
| --- | --- | --- | --- | --- | --- | --- |
| AC007620.3 | 0.940 | 1.000 | -2.850 | 1.000 | 0.000 | 0.012 |
| ACO2 | 0.136 | 0.949 | 0.847 | 1.000 | 0.074 | 0.034 |
| ANKRD22 | 0.491 | 1.000 | 1.043 | 1.000 | 0.000 | 0.024 |
| BET1 | 0.121 | 0.955 | 0.866 | 1.000 | 0.078 | 0.029 |
| CCP110 | 0.283 | 0.985 | 0.954 | 1.000 | 0.042 | 0.029 |
| CD274 | 0.875 | 1.000 | 1.080 | 1.000 | 0.000 | 0.012 |
| CERCAM | 0.586 | 1.000 | 1.016 | 1.000 | 0.000 | 0.015 |
| CH507.42P11.8 | 0.431 | 1.000 | 1.004 | 1.000 | 0.000 | 0.019 |
| CHIT1 | 0.023 | 0.972 | 0.917 | 0.999 | 0.120 | 0.029 |
| CIB2 | 0.252 | 0.985 | 0.954 | 1.000 | 0.049 | 0.030 |
| DARS-AS1 | 0.420 | 1.000 | 1.094 | 1.000 | 0.000 | 0.029 |
| EEFSEC | 0.024 | 0.879 | 0.638 | 1.000 | 0.119 | 0.042 |
| FAM228B | 0.701 | 1.000 | 1.119 | 1.000 | 0.000 | 0.018 |
| GBP1P1 | 0.845 | 1.000 | 1.080 | 1.000 | 0.000 | 0.013 |
| GBP5 | 0.820 | 1.000 | 1.081 | 1.000 | 0.000 | 0.014 |
| HBEGF | 0.300 | 0.989 | 0.968 | 1.000 | 0.038 | 0.028 |
| LARS2 | 0.666 | 1.000 | 1.044 | 1.000 | 0.000 | 0.014 |
| MCM8 | 0.487 | 1.000 | 1.009 | 1.000 | 0.000 | 0.017 |
| MLKL | 0.432 | 1.000 | 1.007 | 1.000 | 0.000 | 0.024 |
| NAGS | 0.556 | 1.000 | 2.139 | 1.000 | 0.000 | 0.023 |
| NEK11 | 0.284 | 0.988 | 0.964 | 1.000 | 0.042 | 0.026 |
| NME7 | 0.922 | 1.000 | 1.104 | 1.000 | 0.000 | 0.010 |
| PNISR | 0.193 | 0.928 | 0.784 | 1.000 | 0.061 | 0.032 |
| RP11.329N15.3 | 0.128 | 0.973 | 0.918 | 1.000 | 0.077 | 0.033 |
| RP11.366L5.1 | 0.509 | 1.000 | 1.045 | 1.000 | 0.000 | 0.022 |
| RP11.43F13.1 | 0.367 | 0.999 | 0.998 | 1.000 | 0.019 | 0.019 |
| RP5.1065J22.8 | 0.548 | 1.000 | 1.036 | 1.000 | 0.000 | 0.018 |
| SCD5 | 0.563 | 1.000 | 1.011 | 1.000 | 0.000 | 0.013 |
| SP110 | 0.836 | 1.000 | 1.046 | 1.000 | 0.000 | 0.013 |
| TERF1 | 0.106 | 0.943 | 0.830 | 1.000 | 0.082 | 0.033 |
| USP18 | 0.972 | 1.000 | 1.049 | 1.000 | 0.000 | 0.005 |
| ZNF292 | 0.304 | 0.992 | 0.977 | 1.000 | 0.037 | 0.026 |

**Figure S1.** **Flowchart of RNA selection.** The figure summarizes the filtering steps to select the RNAs of interest for the longitudinal analyses; the number of excluded RNA at each step is reported along with the reason for exclusion.


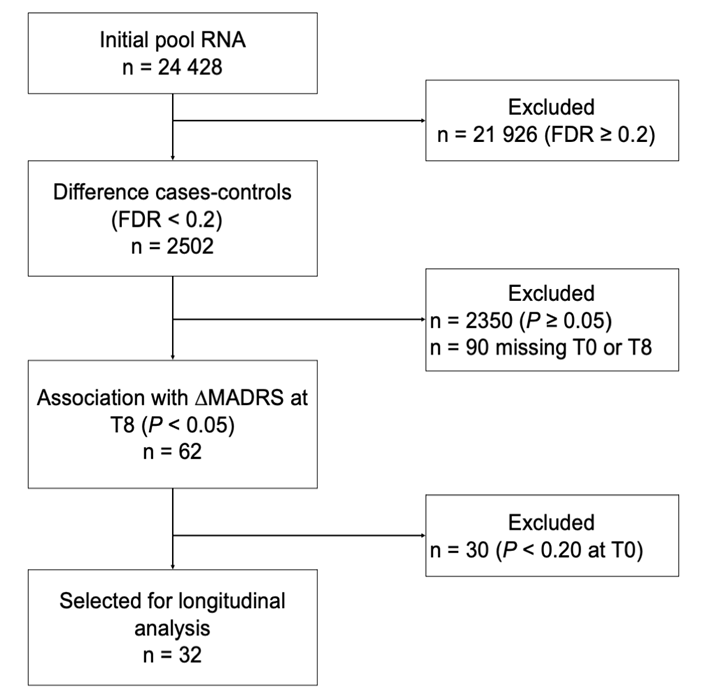


**Figure S2: Expression of target genes in neural progenitor cells following drug treatments.** The effects of drug treatment on gene expression was assessed after 48 hours of treatment, for CERCAM (**A**), DARS-AS1 (**B**), FAM228B (**C**), and HBEGF (**D**). Expression values were normalized to B-actin, and error bars represent SEM.
